# Raising Children

**Published:** 1863-09

**Authors:** 


### RAISING CHILDREN.

I HAVE never lost a child, and it seems to me, if one of my four were to die, the sun would never shine to me again; at least, never as brightly as before; and that a pall would hang over all that made life desirable. I know that time soothes every sorrow, and takes off the sharp edge of the most heart-crushing afflictions; but still, sad thoughts would hover mournfully over the departed; coming unbidden even in the press of business; on the crowded street; in the gay assembly.

Next to the great calamity of a lost child, is the torture of having one before your eyes every day of your existence the victim of some slow disease, of some incurable malady, of some eating, painful, fatal ailment, or some distressing or humiliating deformity. All my children were born apparently delicate, except the last; they seemed to me like wax-work; almost too frail to be handled; but all were free from blemish or deformity. Two things were in their favor, they were born of healthy parents, and hadn't much sense; of which latter I have always felt particularly glad, in view of the fact that the "smarter" a child is, the brighter its intellect, the more certain it is to die early of brain disease; if not, the chances are that the intellect will wane early; will not answer the expectations formed of it; or if it does, and practical life is reached, genius and magnificent abilities do not add to the probabilities of domestic happiness of success in life, or a very enjoyable existence.

Genius is erratic, whether in man or woman; and is always precipitating its possessor into all sorts of "fixes," of the unpleasurable kind; geniuses, magnificent minds, generally die drunk or mad; die early, from the effects of opium, liquor or tobacco. My own personal observation bears me out in the saying, that persons of moderate mental caliber, of medium capacities, are most likely to live long, live healthfully, live happily, and live successfully, whether as to making a comfortable living, or having a solid influence in society.

It may be suggestive to remark, in passing, how singularly children "take after," and don't take after their parents, one or both. Of my four, who are between the ages of nine and sixteen, none take after their parents, in what is called smartness; that quality having been monopolized by their father and mother; while all of them are good-looking, and are growing up to be comely, a quality which neither parent ever pretended to, unless in a fit of insanity. But the good health of the parents has descended to all the children; the measure of it may be expressed by the fact, that from the first day of September last, until this present first day of August, neither of the four has lost a meal from sickness; and all four have not lost half a dozen days from school, during the ten months' session. No kind of wind or weather, rain, hail, sleet, or snow, has been allowed as an excuse for not going to school, which is just one mile and three quarters from their home; this may be more fully appreciated when it is remembered that to reach school by nine, requires to breakfast at seven of a winter's morning, which must be done by gas-light. "Father," said our little Alice, the other day, as we were taking our morning walk toward school, "I have been going to school three years, and have never been late once"—the word "late" meaning not being there in time to go into the recitation-room precisely at nine. All are required to be at the breakfast-table not later than seven in mid-winter, without being called, or "rung up"—this throws them on their own resources, makes them self-reliant, and is not a hardship, for there is a clock, as well as a thermometer, in every occupied room in the house; and when a routine is once fallen into, which is shown to be convenient and advantageous to all the household, it becomes almost as easy to do right as wrong; besides, when a thing is looked upon as having to be done as a matter of course, it is no longer the burden or hardship that it might at one time have been considered to be. And it is greatly to be regretted that this idea is not better understood in families.

These details, and others to be mentioned, are not given as proof positive, that the healthfulness of my children is the result of my management; for there may have been quite as much health in other families of the same size, age, and general circumstances; still, some of the observances must have contributed more or less to such a gratifying result; and it is certainly a great happiness to have not an hour's sickness in a growing family, for months and years together. As to eating, breakfast is taken at seven o'clock the year round, a plain luncheon is carried to school, to be eaten at twelve M.; they are dismissed at three, and by four o'clock, sit down to dinner, and nothing more is allowed until breakfast next morning; this arrangement for eating was adopted as the best under the circumstances, in connection with the school; otherwise, the old-fashioned plan of early breakfast, dinner at noon, and supper in the evening, seems to be the most rational and convenient. All eating, except at breakfast, school-lunch at noon, and dinner at four o'clock, is discouraged, because it occasions unnecessary trouble in the house; but more than all, to prevent that irregularity of eating at regular meals, which is an inevitable result of a permission to eat between times. Besides, if a child is allowed to eat between meals, he is very sure some times to eat so heartily as not to be much hungry at the following regular meal, the result soon being that he is hungry only between meals, while at the regular hours for eating with the family, he is merely a nibbler, and soon gets to eating half a dozen times a day, and at no one time much; the result is, there is no real vigorous appetite for solid substantial food, consequently the strength is not sustained; instead of running about the house cheerily, he sits, and lolls, and mopes, and lounges about in listless indifference and fretfulness, which in turn grows to a settled unloveliness, throwing a cloud over the whole family. But this is not all; it is known to be a fact, that three fourths of all who die of consumption, trace the beginnings of their troubles to their "teens." And it is very naturally brought on thus: irregular eating induces irregular bodily functions; takes away the vigorous appetite, and, as above stated, ends in an indisposition to exercise; this, in turn, induces debility of body and lassitude of mind. Such persons always take cold easily; easily fall into any kind of sickness which may happen to prevail, simply from a want of vigor to repel the most ordinary causes of diseases; hence, as it requires but a little to make them sick, they are almost all the time ailing in some way or other, and eventually become confirmed invalids; the sons thereby to become discouraged; and the daughters, if they live to marry, need nursing from the beginning; are unfit to keep house; are a constant drag to the husband, instead of an aid in becoming thrifty and prosperous; and all ending too often in domestic indifference, or a feeling of discouragement, venting itself in the expression, "It isn't worth while for me to try and lay up any thing;" and when a man arrives at such a conclusion, he is practically lost to society as far as becoming a prosperous and influential business man is concerned.

But if a child by frequent eating comes to the table with but an indifferent appetite, an inevitable result is, that it has no relish for plain, substantial, nourishing food; it don't like this, and don't want that, and objects to the other usual stand-bys on the table; then comes the complaint, that this "isn't good," and that "an't nice;" and the foundation is laid for that hatefully fretful, complaining, repining habit, which in a man is contemptible, and in woman a degradation.

Nor is this the only evil of so arranging the eating that children shall very often come to the table without a vigorous appetite, for as soon as the mother observes it, she becomes apprehensive that the child is about to be sick, and that if it does not eat, it will become weak and soon be confined to bed; and her imagination running riot, lays the child in the grave in a few days; in order to prevent this, she reasons thus: when the child was well it ate heartily, and if it can be got to eat heartily it will be well again; therefore she sets her wits to work to get up various delicacies to tempt the child to eat heartily, and as a matter of course precipitates an unfavorable result, by forcing food on the system in a measure, when it is not called for, thus oppressing the vital powers more and more, and prematurely exhausting the recuperative energy; making it absolutely more certain that the child will get really sick, and if it does, proportionably diminishes the chances of recovery. The true plan, most especially with children, and one which would avert, at the very least, one half of their sickness is, the very moment the appetite is noticed to be not so good as it was, compel the child to take one half less than it really is inclined to; thus, by diminishing the labor of the stomach, it has a chance to rest, to recover its energy, when the appetite begins at once to return, and the child is well. A grand rule would this be for persons of all ages, but it takes a man of force of character to do this; the pampered, the self-indulgent, the undecided, feeble-minded folk are altogether inadequate to such a feat of moral courage. Many an attack of illness might be warded off from children by the exercise of a very little attention and firmness. If a child wakes up in the morning and calls for a drink of water the first thing, such child is perfectly certain to be sick before noon. The course to be pursued, is to keep him in bed, and by warm drinks promote perspiration, eating nothing whatever until the afternoon, when he may amuse himself by nibbling at some cold, dry bread, and the next day he will be about again; otherwise, a breakfast will be eaten, fever comes on, vomiting, and several days' illness.

If a child is allowed to eat what he wants at supper, he will in less than a week have very little appetite for breakfast; the next step is to feel hungry two or three hours later; this again interferes with the appetite for dinner, and in a short time all system and regularity in eating is destroyed, inducing inevitably and always general ill-health. Mothers generally are incapable of exercising the requisite degree of firmness, in compelling their children to take light suppers — that is, a cup of warm drink and a piece of cold bread and butter. The easiest way to bring this about, is to never have any thing else on the table, and whenever children, as they certainly will, until better trained, exhibit any dissatisfaction, by word or sign, or expression of countenance, on entering the supper-room, let it be met with a simple and quiet requisition to go at once to bed, without any supper at all.

With the ordinary routine of provision on the table, it is well to let children eat as much as they want; they will seldom take too much for breakfast or dinner, but when a new dish comes on the table, whether one just learned from a neighbor, or the first of the season, any child that seems to be particularly fond of it, should be restricted to a small amount, which may be gradually increased from day to day; otherwise, the child is very certain to gorge himself, be sick, and then never like that dish afterward. It is an inexcusable tyranny to require children to eat or drink what they have no relish for; better let them consult their instincts. We have only to appreciate the unreasonableness of this forcing process, and its hardship, by trying to compel ourselves to swallow what we have no relish for.

Most parents find a constantly recurring difficulty in getting their children off to bed in season at night; all of them have a disinclination to retiring early. But it is of the utmost importance to make an iron rule in the household in that respect, at least as to every child going to school. There can be no health without it, for two reasons: the eyes will soon become inflamed and sore, and by not getting sleep enough, the brain does not work with activity; it takes hold of the lessons with reluctance, as it were, all study becomes a bore, the child falls behind, or, in his efforts to keep up with the class, especially if a girl, brain-fever or some other malady supervenes, and days and weeks are lost at school, and sometimes even life itself. Children should be required to go to bed at such an early hour that they may wake up of themselves in the morning; this is an indication that they have had all the sleep that nature can take; then they are lively, cheerful, and hilarious all day; but if, from having company, or being out at "meeting," parties, or amusements, they are kept out of bed an hour or two later than usual, they will wake up about the accustomed time, but they are pretty sure to come to the breakfast-table with unbuoyant countenances; there are frowns instead of fun and smiles, and they are very apt to be fretful, captious, or complaining for the whole day afterward. Let it be arranged the year round, that school-children shall wake up at daylight; this will not only prevent the necessity of ruining the eyes by night-study, but also the more injurious practice of studying by artificial light in the morning. Several of the associates of our daughters have permanently weak eyes, yellow matter constantly about the eye-lashes, in consequence of their sitting up to ten and eleven o'clock at night at their books, and every once in a while they are "absent," on the ground of having "sore eyes." We have known two cases of late, where children of thirteen were allowed to sit up until eleven o'clock at night, with the full glare of gaslight falling on the bright white page; in a very short time they had to lose from one to three weeks. In one case, a girl was required to go to school, day after day, when her eyes were in such a state from night-study, that she could not use them at all, but was merely a listener to the recitations; a barbarity on the part of teacher and parents of which a savage heart should be ashamed. If parents would systematically attend to one point in reference to their children who are attending school, in addition to regularity in eating, and sleeping to the utmost that nature will take, it would avert an infinite amount of ill from their children in the course of a lifetime; it is simply this, let breakfast be taken sufficiently early to allow them perfect leisure to attend to all the calls of nature before they leave for school. It is perfectly certain that multitudes of children, and even grown persons, lay the foundation for life-long diseases and sufferings, in consequence of neglecting to attend systematically to this suggestion.

